# Supplementary material for: An integrated transcriptome mapping the regulatory network of coding and long non-coding RNAs provides a genomics resource in chickpea
Source: Commun Biol. 2022 Oct 19;5:1106. doi: 10.1038/s42003-022-04083-4 (PMC9581958; doi:10.1038/s42003-022-04083-4)
Supplement: Supplementary file 2 — Supplementary Information [file 42003_2022_4083_MOESM2_ESM.pdf]

SUPPLEMENTARY INFORMATION

Supplementary Table 1. Summary of RNA sequencing (Iso-seq) data generated using PacBio platform.

**Library fragment size**	**Number of consensus reads**	**Number of full-length non-chimeric reads**
0.8 – 2 kb	236206	140137
2 – 3 kb	310563	192631
3 – 6 kb	954218	472143
5 – 10 kb	637395	205319
Total	2138382	1010230

Supplementary Table 2. Summary of transcriptome assemblies generated from PacBio and Illumina sequencing data and final consensus reference transcriptome assembly.

	**PacBio transcripts**	**Illumina assembly**	**Total transcripts**	**Unique transcripts/loci (reference transcriptome assembly)**
Total sequences	81269	55313	111620	38818
Total size (Mb)	213.84	109.53	270.17	87.69
Minimum length (bp)	200	200	200	200
maximum length (bp)	7235	19825	19825	19825
Average length (bp)	2631.29	1980.18	2420.42	2259.01
Median length (bp)	2534	1761	2187	1854
N50 length (bp)	3312	2482	3192	3292

Supplementary Table 3. Comparison of chickpea transcriptome assemblies available in the public domain with reference transcriptome assembly.

	**RTA[1]**	**Hybrid assembly[2]**	**RefTrans v1 (2015)[3]**	**RefTrans v2 (2017)[4]**
Total sequences	38818	34760	50864	56176
Total size (Mb)	87.69	35.47	51.03	75.62
Minimum length (bp)	200	100	201	297
maximum length (bp)	19825	15661	15754	16908
Average length (bp)	2259.01	1020.39	1003.27	1346.17
Median length (bp)	1854	735	614	973
N50 length (bp)	3292	1671	1660	1925
N75 length (bp)	2050	1013	814	1093
N90 length (bp)	1156	537	405	610

[1]Reference transcriptome assembly (RTA) (this study)
[2]Transcriptome assembly generated using Illumina and Roche 454 data (Garg et al., 2011, Plant Physiol)
[3]Transcriptome assembly available at Pulse Crop Database (https://www.pulsedb.org/analysis/130)
[4]Transcriptome assembly available at Pulse Crop Database (https://www.pulsedb.org/analysis/161)

Supplementary Table 4. Conservation of lncRNAs in different plant species.

		Total lncRNAs in database		Number of chickpea lncRNAs showing significant similarity		
**Plant species**	**Family**	**GREENC**	**CANTATAdB**	**GREENC**	**CANTATAdb**	**Unique**
Chenopodium_quinoa	Amaranthaceae		17526		8	8
Amborella_trichopoda	Amborellaceae	5698	5511	5	6	11
Spirodela_polyrhiza	Araceae	2111		1		1
Ostreococcus_lucimarinus	Bathycoccaceae	501		3		3
Arabidopsis_lyrata	Brassicaceae	4363	7593	8	0	8
Arabidopsis_thaliana	Brassicaceae	3008	4373	10	7	10
Brassica_napus	Brassicaceae		12010		6	6
Brassica_oleracea	Brassicaceae		7338		3	3
Brassica_rapa	Brassicaceae		8501		4	4
Capsella_grandiflora	Brassicaceae	1394		3		3
Capsella_rubella	Brassicaceae	1458		9		9
Eutrema_salsugineum	Brassicaceae	1669		9		9
Ananas_comosus	Bromeliaceae	3376	10404	3	2	4
Carica_papaya	Caricaceae	3987		3		3
Chlamydomonas_reinhardtii	Chlamydomonadaceae	619	3425	0	0	0
Coccomyxa_subellipsoidea_C-169	Coccomyxaceae	668		0		0
Cucumis_sativus	Cucurbitaceae	1929	7348	7	9	13
Manihot_esculenta	Euphorbiaceae	3468	9504	13	8	17
Ricinus_communis	Euphorbiaceae	4198		7		7
Glycine_max	Fabaceae	6689	3096	109	19	123
Medicago_truncatula	Fabaceae	9676	3590	176	65	230
Phaseolus_vulgaris	Fabaceae	1946		51		51
Trifolium_pratense	Fabaceae		10179		136	136
Physcomitrella_patens	Funariaceae	9690	1498	2	0	2
Galdieria_sulphuraria	Galdieriaceae		1917		2	2
Chondrus_crispus	Gigartinaceae		224		0	0
Linum_usitatissimum	Linaceae	2598		2		2
Corchorus_capsularis	Malvaceae		6459		13	13
Gossypium_raimondii	Malvaceae	4216		15		15
Theobroma_cacao	Malvaceae	4268	5256	14	1	15
Micromonas_pusilla_CCMP1545	Mamiellaceae	651		3		3
Micromonas_pusilla_RCC299	Mamiellaceae	351		0		0
Musa_acuminata	Musaceae	4071	3001	4	3	6
Eucalyptus_grandis	Myrtaceae	4639		8		8
Mimulus_guttatus	Phrymaceae	2333		10		10
Brachypodium_distachyon	Poaceae	5584	4945	3	5	7
Hordeum_vulgare	Poaceae		7970		5	5
Leersia_perrieri	Poaceae		6402		3	3
Oryza_barthii	Poaceae		7062		10	10
Oryza_brachyantha	Poaceae		6004		2	2
Oryza_nivara	Poaceae		8955		3	3
Oryza_punctata	Poaceae		3459		2	2
Oryza_rufipogon	Poaceae		10261		9	9
Oryza_sativa_Japonica_Group	Poaceae	5237	2788	0	4	4
Setaria_italica	Poaceae	3492	4208	1	1	2
Sorghum_bicolor	Poaceae	5305	2600	2	3	5
Triticum_aestivum	Poaceae	38820		5		5
Zea_mays	Poaceae	18110	10761	8	3	11
Fragaria_vesca	Rosaceae	3503		3		3
Malus_domestica	Rosaceae	4126	10924	4	10	13
Prunus_persica	Rosaceae	3301	2902	20	3	21
Citrus_clementina	Rutaceae	2192		6		6
Citrus_sinensis	Rutaceae	2562		5		5
Populus_trichocarpa	Salicaceae	5569	4322	18	9	25
Selaginella_moellendorffii	Selaginellaceae	906	2267	2	2	3
Solanum_lycopersicum	Solanaceae	3440	4716	8	3	9
Solanum_tuberosum	Solanaceae	6680	5790	5	5	9
Vitis_vinifera	Vitaceae	2526	4542	6	6	12
Volvox_carteri	Volvocaceae	1134		0		0
Zostera_marina	Zosteraceae	1393		1		1

Supplementary Table 5. Members of TF families identified in chickpea, Arabidopsis and soybean.

TF Family	Chickpea	Arabidopsis	Soybean	TF Family	Chickpea	Arabidopsis	Soybean
AP2-EREBP	180	145	303	ARR-B	16	14	25
MYB	143	165	303	LIM	16	13	33
bHLH	131	138	256	Tify	16	15	40
HB	125	93	204	ARID	14	10	14
CCHC	106	28	94	BSD	13	11	20
WRKY	99	72	159	SAP	12	23	47
NAC	87	112	171	RWP-RK	12	14	19
bZIP	83	73	125	IWS1	12	9	21
C3H	78	51	92	CAMTA	11	6	10
CCAAT	75	66	150	EIL	11	6	15
SNF2	73	43	65	CPP	10	8	7
GRAS	71	34	116	C2C2-YABBY	9	6	17
mTERF	66	35	53	Sigma70-like	9	6	15
G2-like	60	42	82	C2C2-CO-like	8	16	13
MADS	60	108	157	GeBP	8	22	11
Trihelix	50	31	76	BES1	7	8	19
ABI3VP1	48	68	45	GRF	7	9	12
MYB-related	47	41	110	SRS	7	11	21
LOB	46	43	79	SWI/SNF-SWI3	7	5	7
AUX/IAA	45	29	82	CSD	6	4	6
SET	45	42	65	E2F-DP	6	8	14
Alfin-like	42	36	55	PBF-2-like	6	3	9
TRAF	41	22	36	TAZ	6	8	5
C2C2-Dof	39	36	70	DDT	6	2	7
Bromodomain	37	27	44	DBP	5	5	13
ARF	33	23	58	NOZZLE	5	8	6
FHA	29	17	28	Rcd1-like	5	3	4
FAR1	29	17	66	VARL	4	2	6
GNAT	29	32	63	S1Fa-like	3	3	5
C2C2-GATA	28	30	51	ULT	3	3	13
C2H2	28	33	66	VOZ	3	2	7
Jumonji	28	17	40	TIG	3	0	1
SWI/SNF-BAF60b	28	17	31	Coactivatorp15	3	4	6
PHD	27	27	31	HRT	2	2	2
HSF	26	24	46	LFY	2	1	4
SBP	25	17	39	BBR/BPC	2	7	14
TCP	25	24	53	MBF1	2	3	3
zf-HD	21	17	47	Pseudo_ARR-B	2	5	11
HMG	20	11	25	MED6	2	1	1
PLATZ	19	12	22	MED7	2	2	2
TUB	19	11	26	RB	1	1	3
OFP	19	17	35	SOH1	1	1	2
LUG	17	2	9				

Supplementary Table 6. DNA polymorphisms within the *cis*-regulatory motifs in the promoter regions of QTL-associated differentially expressed transcripts for different traits.

Transcript ID	Motif	Motif sequence	Motif position from TSS	SNP/InDel site (from TSS)	Trait
TC03150	AP1	aCAAAA	-60:-66	-63	100SDW
	PIF4	tCTTTT	-100:-106	-105	100SDW
	AP1	atATATA	-334:-341	-341	100SDW
	SEP3	aGTTGAttctc	-1116:-1127	-1125	100SDW
	HAT22	caATAAT	-1136:-1143	-1143	100SDW
	FLM	cttCTATT	-1266:-1274	-1266	100SDW
	AP1	TTTTGt	-1280:-1286	-1282	100SDW
	AP1	tTCTTC	-1358:-1364	-1360	100SDW
	DREB2A	actctaccatcatttCACGTg	-1441:-1462	-1447	100SDW
	PIF4	tCTTTT	-1516:-1522	-1520	100SDW
	SEP3	tcaACTTT	-1553:-1561	-1556	100SDW
TC03165	BZIP28	CCAATct	-113:-120	-114	100SDW
	SEP3	TTTTGt	-1509:-1515	-1514	100SDW
	AP1	tTTTGGa	-1559:-1566	-1565	100SDW
	AtMYB12	GACGTaga	-1784:-1792	-1786	100SDW
	HSFA6A	aacCTAGAacattct	-1912:-1927	-1918	100SDW
	FHY1	gtTTTTTtttt	-1951:-1962	-1951	100SDW
TC11217	SEP3	ttatTATTTaatgaaa	-826:-842	-826	100SDW
	SEP3	tagagTCAACg	-1243:-1254	-1247	100SDW
TC28764	FLM	AATAGcaa	-69:-77	-72	100SDW
TC34749	LFY	cttattTTTTTcttt	-1839:-1854	-1839	100SDW
TC12789	ABF3	tgtcttctattaTTTTTcatt	-106:-127	-106	drought stress
TC12812	SEP3	cTATTT	-904:-910	-904	drought stress
TC12817	PIF4	GTATAaaa	-254:-262	-254	drought stress
TC12847	MYB3	tgtTAGGT	-703:-711	-703	drought stress
	NFYB2	aTGGGCtt	-972:-980	-972	drought stress
TC13153	AP1	TAACCta	-1380:-1387	-1380	drought stress
TC19197	FHY1	aaaaAAAAAag	-72:-83	-72	drought stress
TC19374	HSFA1A	AACCA	-1369:-1374	-1370	drought stress
TC21982	PIF4	AGTCAttt	-82:-90	-83	drought stress
	FEA4	agaTGACG	-559:-567	-565	drought stress
	SOC1	tTTTGGt	-1003:-1010	-1003	drought stress
	HB7	taATAAT	-1079:-1086	-1083	drought stress
TC34364	BZIP28	tGAAAG	-1022:-1028	-1023	drought stress
TC38803	SOC1	tgTTTGG	-33:-40	-35	drought stress
	DELLA	AACTAgag	-45:-53	-47	drought stress
	NFYC2	tgtgATTGGcg	-176:-187	-176	drought stress
	DELLA	aGAAGC	-689:-695	-691	drought stress
	KAN1	gGAATAt	-1576:-1583	-1582	drought stress
	FLM	TTTGG	-1908:-1913	-1912	drought stress
TC05654	REV	TTTGTtttttaga	-533:-547	-545	saliniy stress
TC05663	SOC1	aTATTT	-1001:-1007	-1003	saliniy stress
	DELLA	TAAAGaag	-1129:-1137	-1130	saliniy stress
	FIE	tTATGAtt	-1571:-1579	-1575	saliniy stress
	FHY3	AAACAtta	-1622:-1630	-1622	saliniy stress
TC05677	SVP	tCCAAT	-36:-42	-42	saliniy stress
	SEP3	taTTTAT	-644:-651	-650	saliniy stress
TC35186	RAMOSA1	acAAATGg	-217:-225	-219	saliniy stress
	RD26	tTTTATtttctcatt	-425:-440	-436	saliniy stress
	SVP	GAAAGa	-617:-623	-623	saliniy stress
	ABF4	AGAAAa	-642:-648	-643	saliniy stress
	FHY1	cTTTTT	-771:-777	-775	saliniy stress
	KAN1	aTATTCa	-1470:-1477	-1471	saliniy stress

Supplementary Table 7. List of candidate transcripts showing differential expression under the salinity stress harboring DNA polymorphisms within the transcript and/or their promoter regions.

Transcript ID	TF family	Transcript type	Description	Chr	QTL coordinate	Reference
TC05652		mRNA	ethylene-dependent gravitropism-deficient and yellow-green-like 2	Ca2	30578328:30587367	Soren et al. 2020
TC05654		mRNA	ethylene-dependent gravitropism-deficient and yellow-green-like 2	Ca2		Soren et al. 2020
TC35186		mRNA	ethylene-dependent gravitropism-deficient and yellow-green-like 2	Ca2		Soren et al. 2020
TC05663		mRNA	Phosphoinositide-specific phospholipase C family protein	Ca2	30704305:30710124	Soren et al. 2020
TC05664		mRNA	Phosphoinositide-specific phospholipase C family protein	Ca2	30711185:30718359	Soren et al. 2020
TC05676	C3H	mRNA	zinc finger (CCCH-type) family protein	Ca2	30864411:30868498	Soren et al. 2020
TC05677	C3H	mRNA	zinc finger (CCCH-type) family protein	Ca2		Soren et al. 2020
TC05785		mRNA	histone mono-ubiquitination 2	Ca2	31907298:31921461	Soren et al. 2020
TC35496		mRNA	poly(A) binding protein 2	Ca5	35975572:36672170	Pushpavalli et al., 2015
TC37565		mRNA	phytoene desaturase 3	Ca5	42519617:42544475	Pushpavalli et al., 2015
TC36669		mRNA		Ca7	46171940:46177433	Pushpavalli et al., 2015

SUPPLEMENTARY INFORMATION

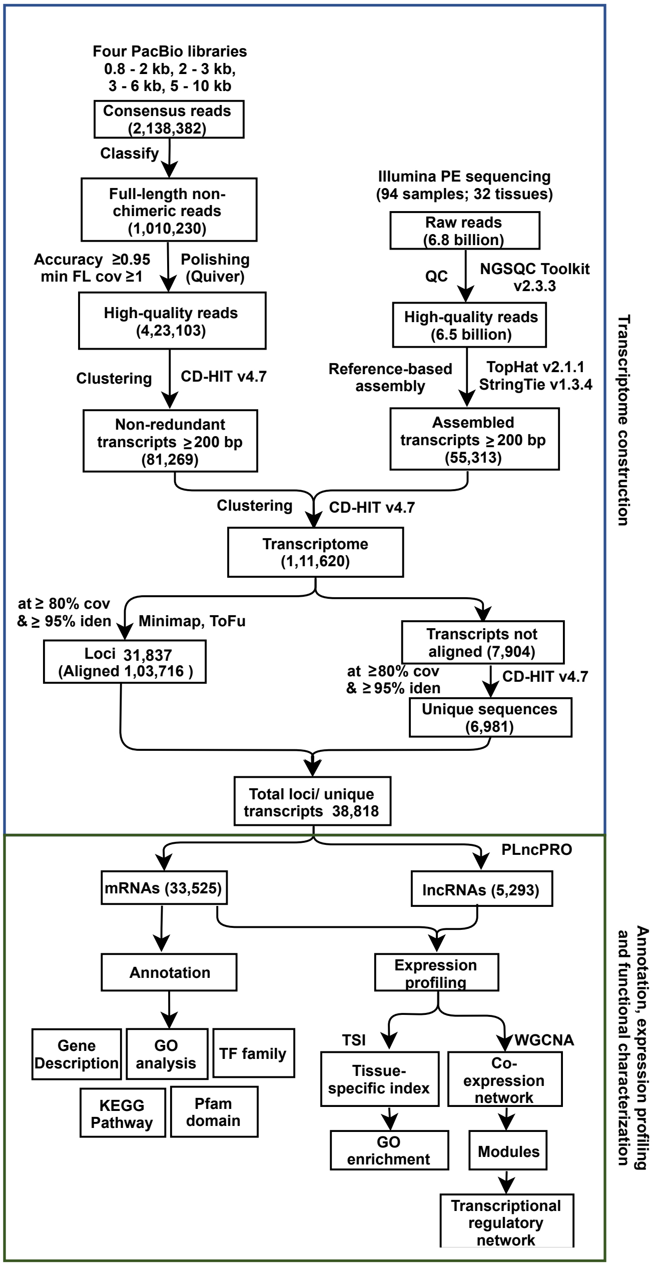

Supplementary Figure 1. An overview of the strategy followed for sequencing, assembly and analyses of the chickpea transcriptome.

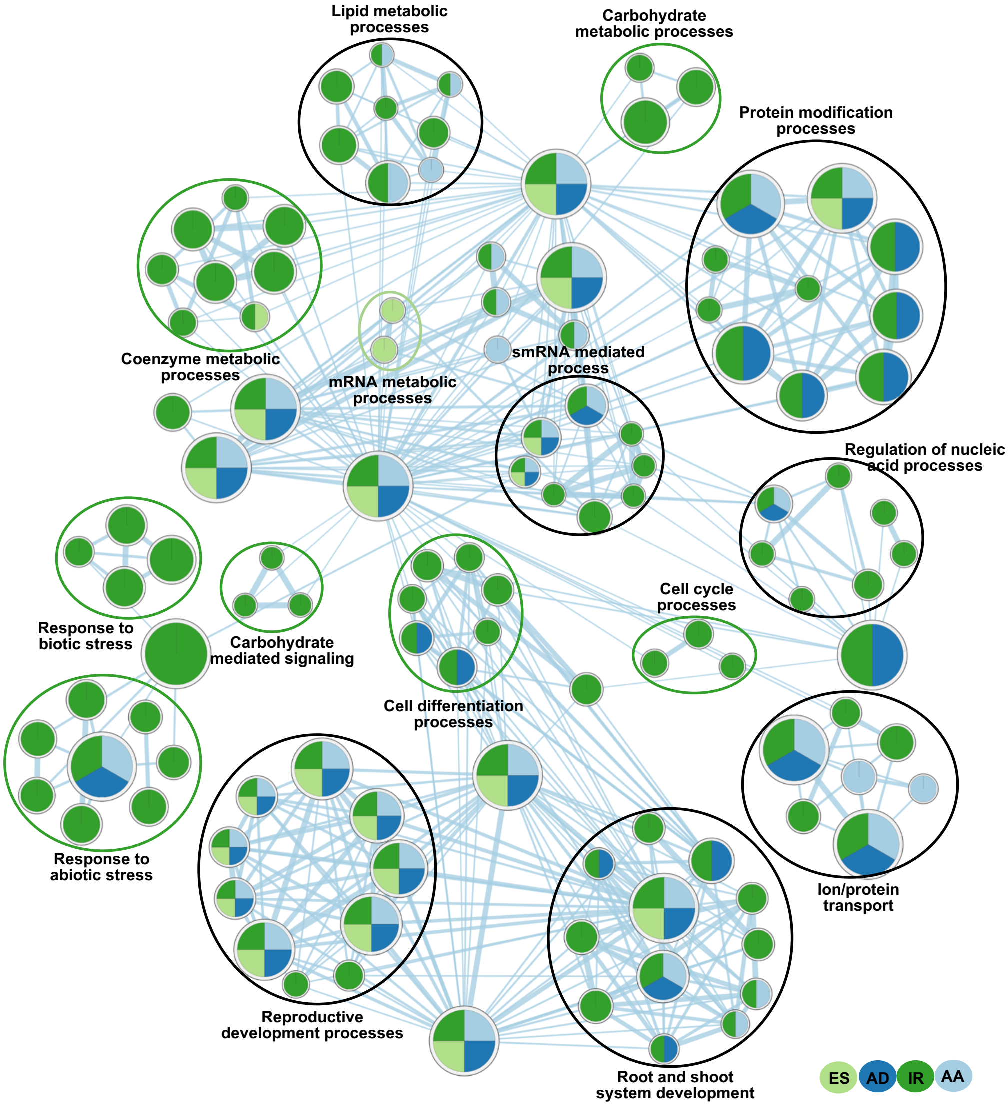

Supplementary Figure 2. Comparative gene ontology (GO) enrichment map analysis of the transcripts representing different types of alternatively spliced isoforms. Comparative GO enrichment maps of the genes representing different types of transcript isoforms, including ES, exon skipping; AD, alternate donor; IR, intron retention and AA, alternate acceptor. Only the significantly enriched (adjusted *P*-value ≤0.01) biological process GO terms are shown.

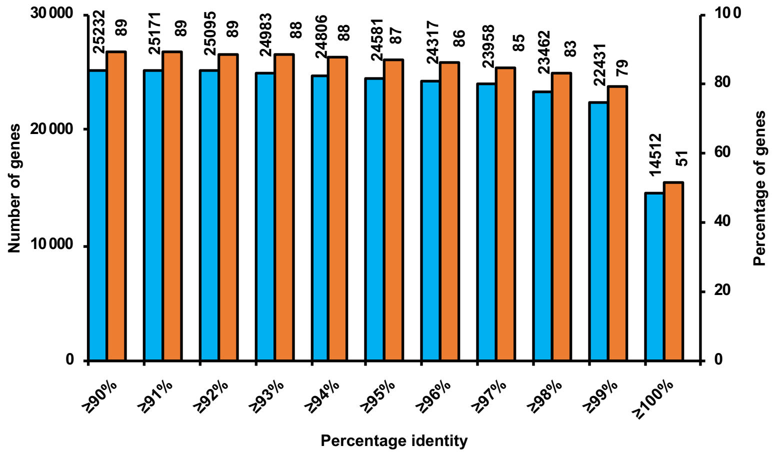

Supplementary Figure 3. Alignment of the genes annotated in the chickpea genome with reference transcriptome assembly. The number/percentage of genes with ≥30% coverage showing different levels of percentage identity with reference transcriptome assembly via BLAT search is shown via bar plot.

(a)

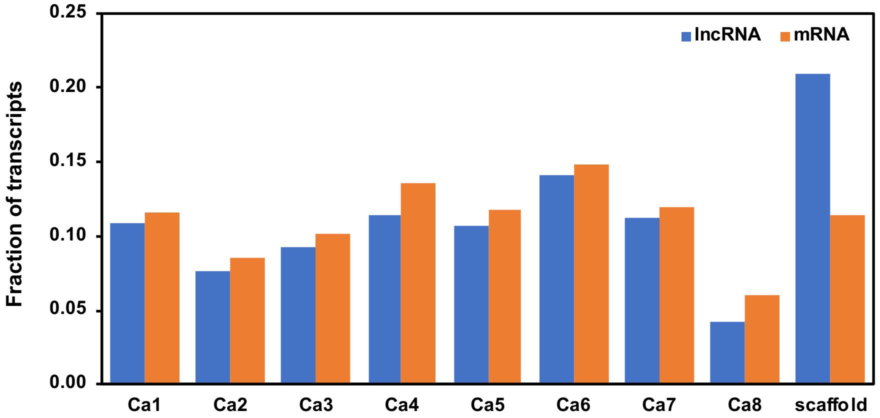

(b)

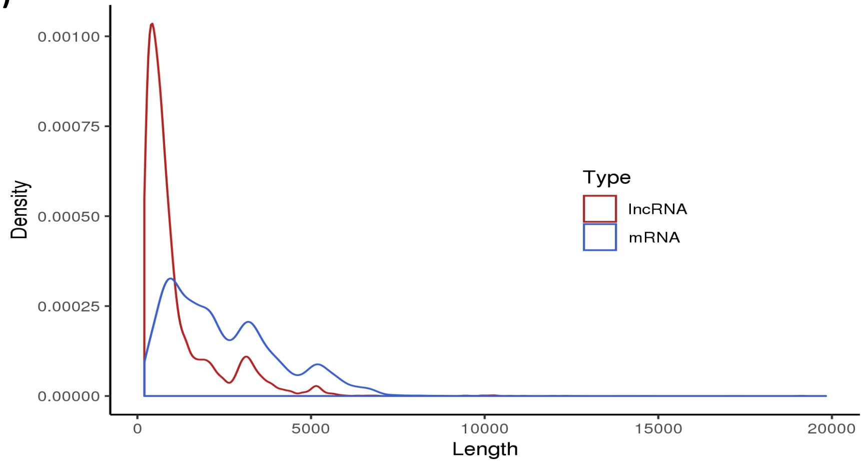

Supplementary Figure 4. Genomic distribution and length distribution of protein-coding genes (mRNAs) and lncRNAs. (a) Bar plot showing the distribution of mRNAs and lncRNAs on chickpea chromosomes. (b) Density plot showing the length distribution of the mRNAs and lncRNAs.

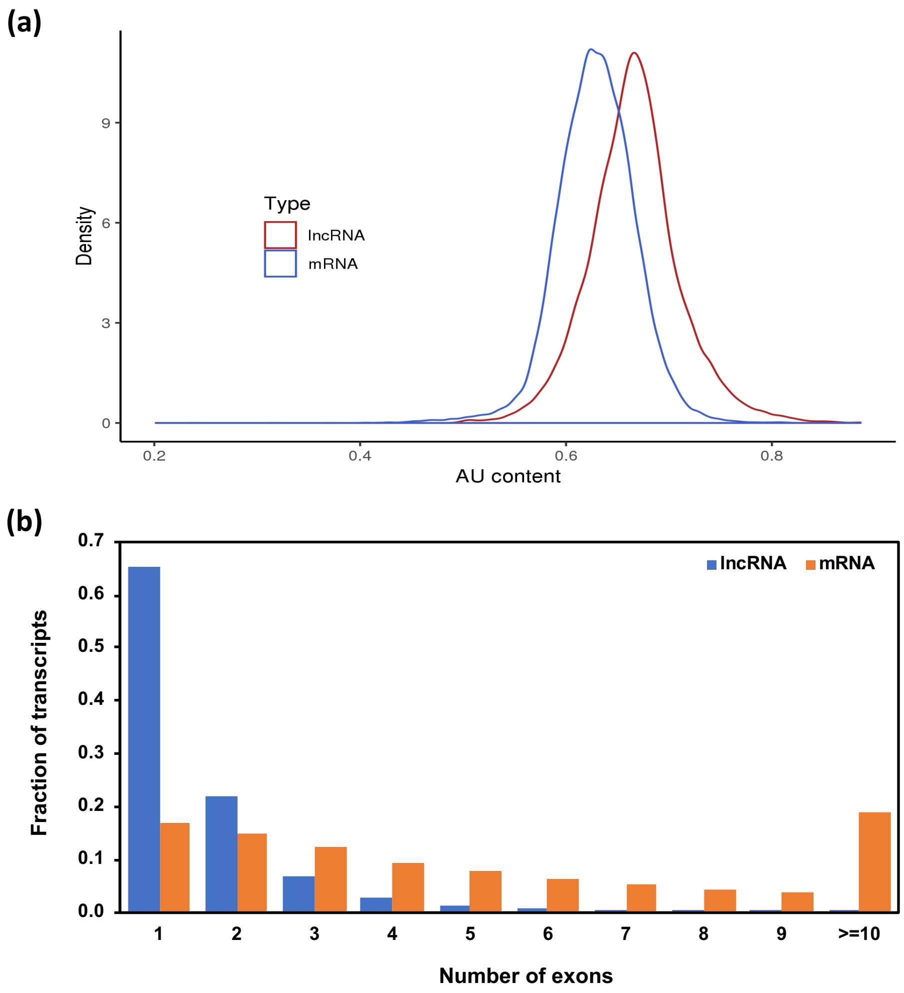

Supplementary Figure 5. AU content and distribution of number of exons in protein coding genes (mRNAs) and lncRNAs. (a) Density plot showing the AU content distribution of the mRNAs and lncRNAs. (b) Bar plot showing the fraction of transcripts harboring different number of exons in the mRNAs and lncRNAs.

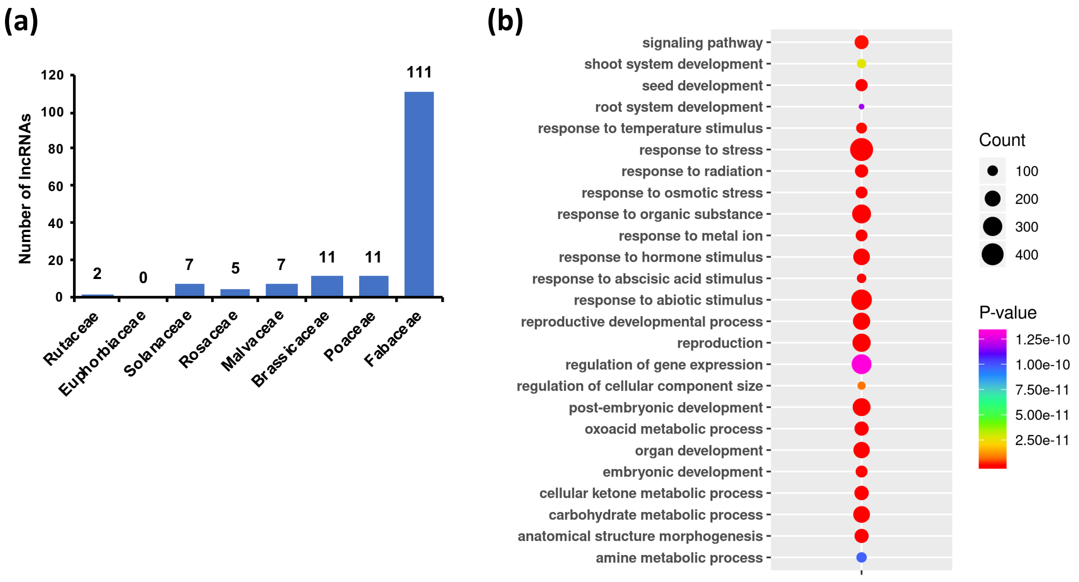

Supplementary Figure 6. Conservation and functional annotation of lncRNAs. (a) Conservation of predicted chickpea lncRNAs across different plant families. The number of lncRNAs showing conservation in at least two plant species of the given families are given. (b) Top 25 enriched gene ontology (GO) biological process terms represented among the lncRNAs. The number of lncRNAs (circle size) with significance *P-value* (color scale) associated with different GO terms are shown.

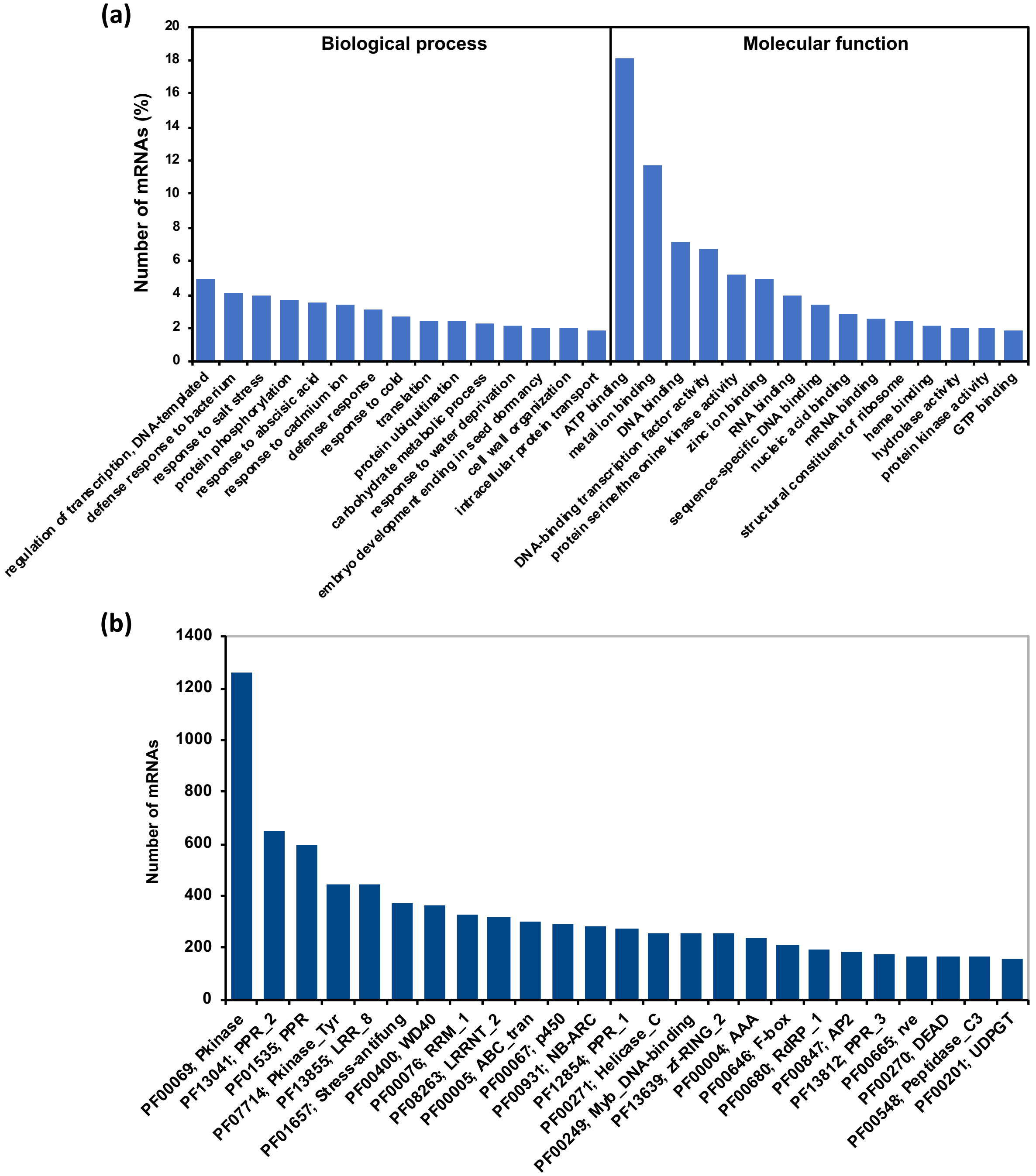

Supplementary Figure 7. Functional characterization of protein-coding mRNAs. (a) GO assignment to the protein-coding genes in the chickpea transcriptome assembly. Top 15 most highly represented biological process and molecular function GO terms are shown. (b) Top 25 PFam domains represented in the protein-coding chickpea transcriptome are shown in bar graph. PFam identifier and name of the domains are given.

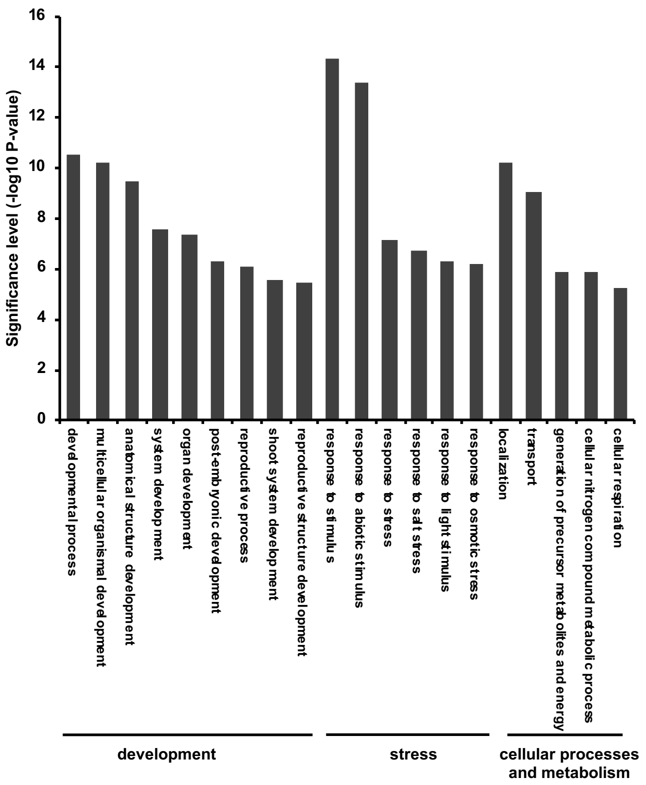

Supplementary Figure 8. GO terms significantly represented among the novel transcripts (13,586) that were either not predicted earlier in the chickpea genome annotation or not represented in the chickpea genome assembly.

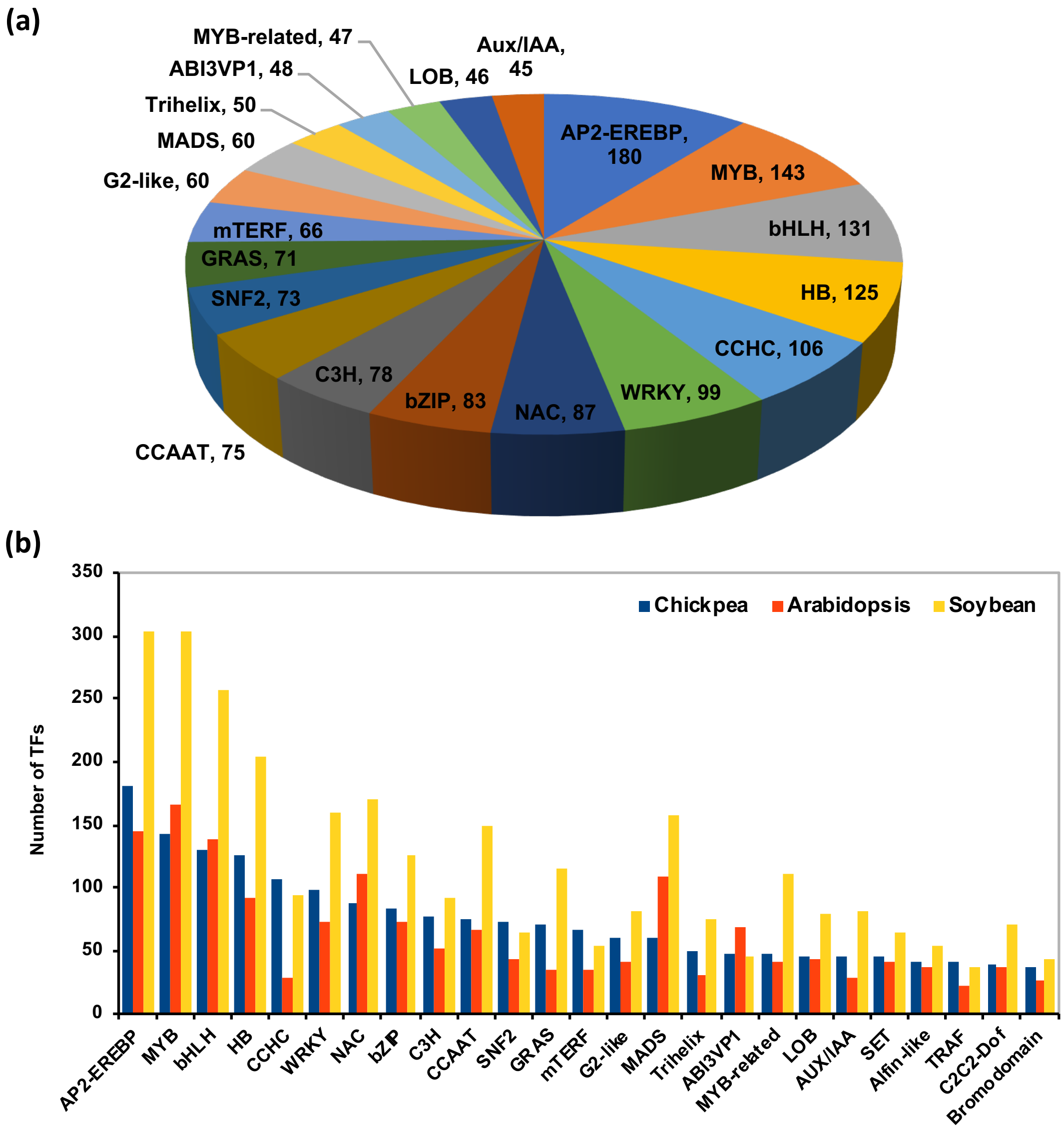

Supplementary Figure 9. Transcription factor families represented in the protein-coding genes in chickpea and comparison with other plants. (a) Top 20 transcription factor families represented in the protein-coding chickpea transcriptome. (b) The number of members of top 25 transcription factor families in chickpea, Arabidopsis and soybean is shown.

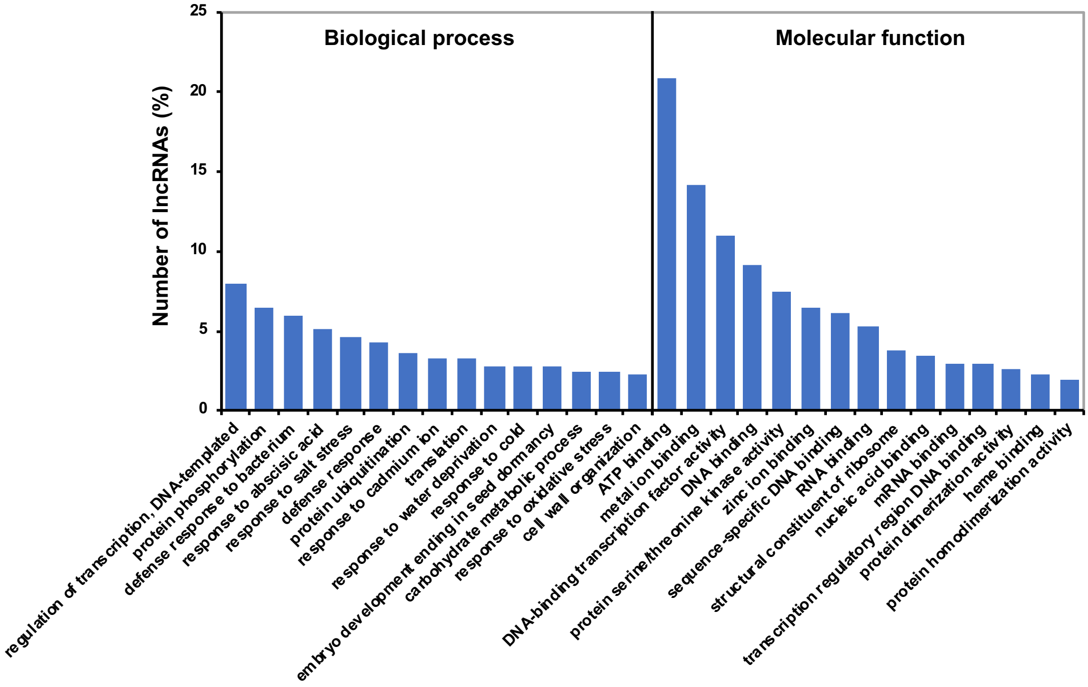

Supplementary Figure 10. GO assignment to the lncRNAs predicted in the chickpea transcriptome. Top 15 biological process and molecular function GO terms associated with lncRNAs are shown.

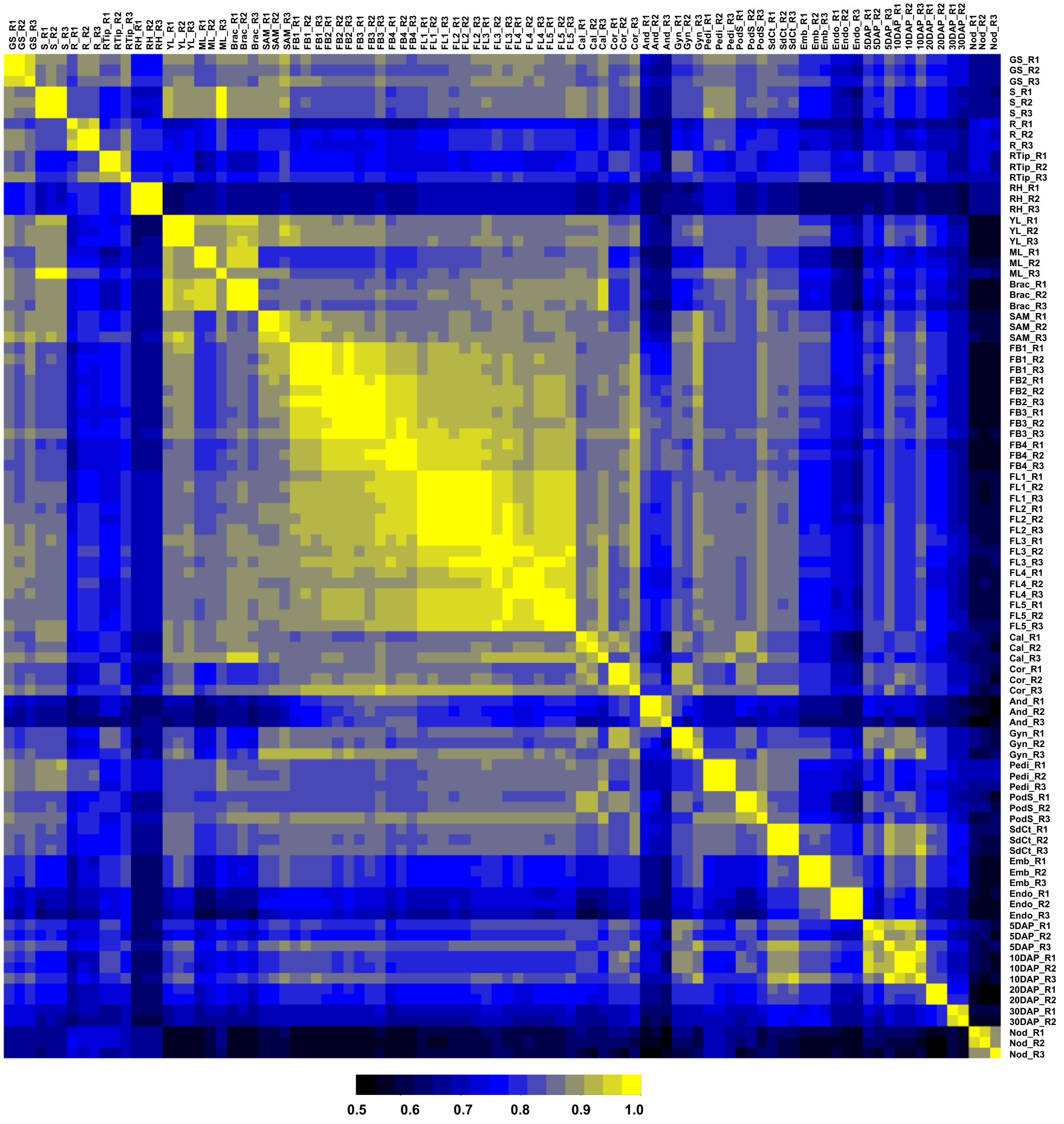

Supplementary Figure 11. Heatmap showing correlation among the biological replicates of all the tissue samples used for RNA sequencing on Illumina platform. GS, germinating seedling; S, shoot; ML, mature leaf; YL, young leaf; Brac, bracteole; R, root; Rtip, root tip; RH, root hair; Nod, nodule; SAM, shoot apical meristem; FB1-FB4, stages of flower bud development; FL1-FL5, stage of flower development; Cal, calyx; Cor, corolla; And, androecium; Gyn, gynoecium; Pedi, pedicel; Emb, embryo; Endo, endosperm; SdCt, seed coat; PodSh, podshell; 5DAP, seed 5 days after pollination; 10DAP, seed 10 days after pollination; 20DAP, seed 20 days after pollination and 30DAP, seed 30 days after pollination. R1, R2 and R3 indicate different biological replicates of each tissue sample. Color scale at the bottom represents Pearson correlation.

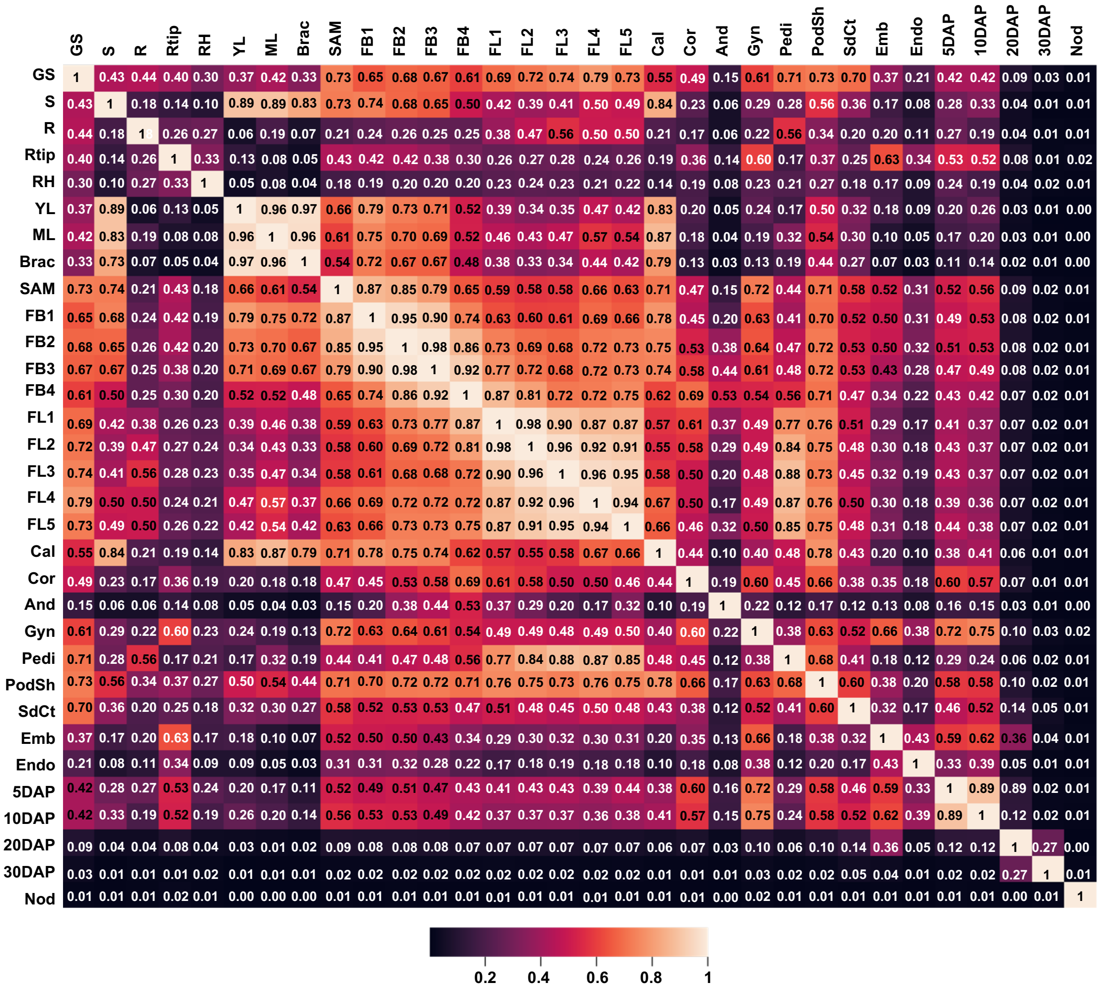

Supplementary Figure 12. Heatmap showing correlation among transcriptomes of all the tissue samples analyzed in this study. GS, germinating seedling; S, shoot; ML, mature leaf; YL, young leaf; Brac, bracteole; R, root; Rtip, root tip; RH, root hair; Nod, nodule; SAM, shoot apical meristem; FB1-FB4, stages of flower bud development; FL1-FL5, stage of flower development; Cal, calyx; Cor, corolla; And, androecium; Gyn, gynoecium; Pedi, pedicel; Emb, embryo; Endo, endosperm; SdCt, seed coat; PodSh, podshell; 5DAP, seed 5 days after pollination; 10DAP, seed 10 days after pollination; 20DAP, seed 20 days after pollination and 30DAP, seed 30 days after pollination. Color scale at the bottom represents Pearson correlation.

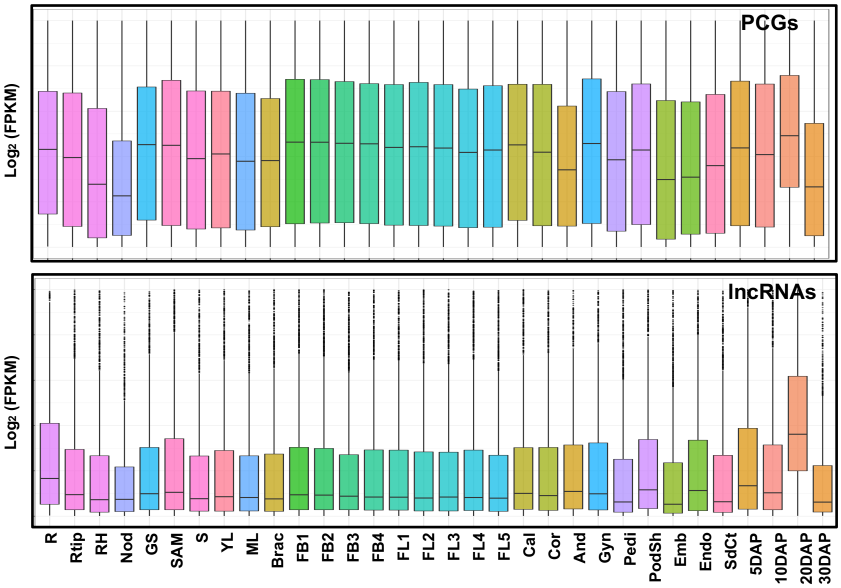

Supplementary Figure 13. Expression patterns of protein-coding genes and lncRNAs in different tissues/organs in chickpea. Percentage of transcription factor (TF) encoding genes, non-TF protein-coding genes (PCGs) and lncRNAs expressed in the 32 tissues/organs analyzed.

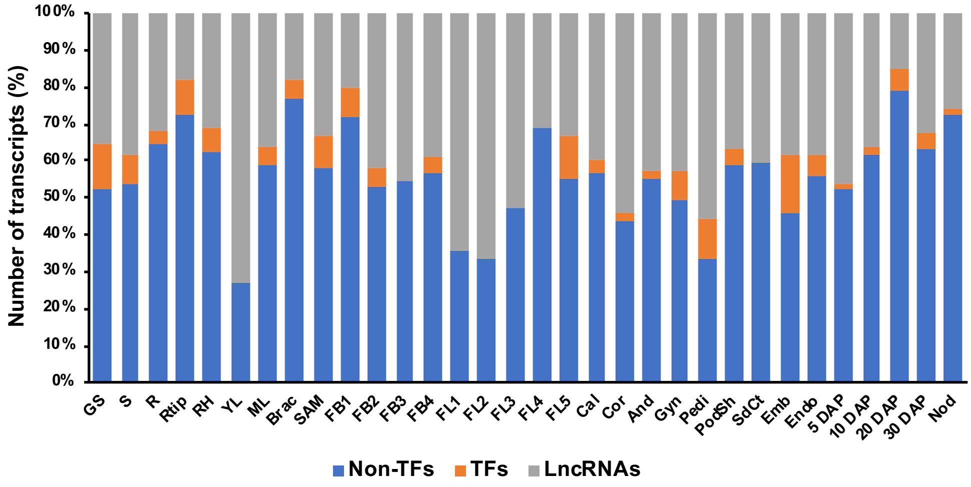

Supplementary Figure 14. Fraction of transcription factor (TF) genes, non-TF genes and lncRNAs represented in tissue-specific transcripts for each tissue/organ.

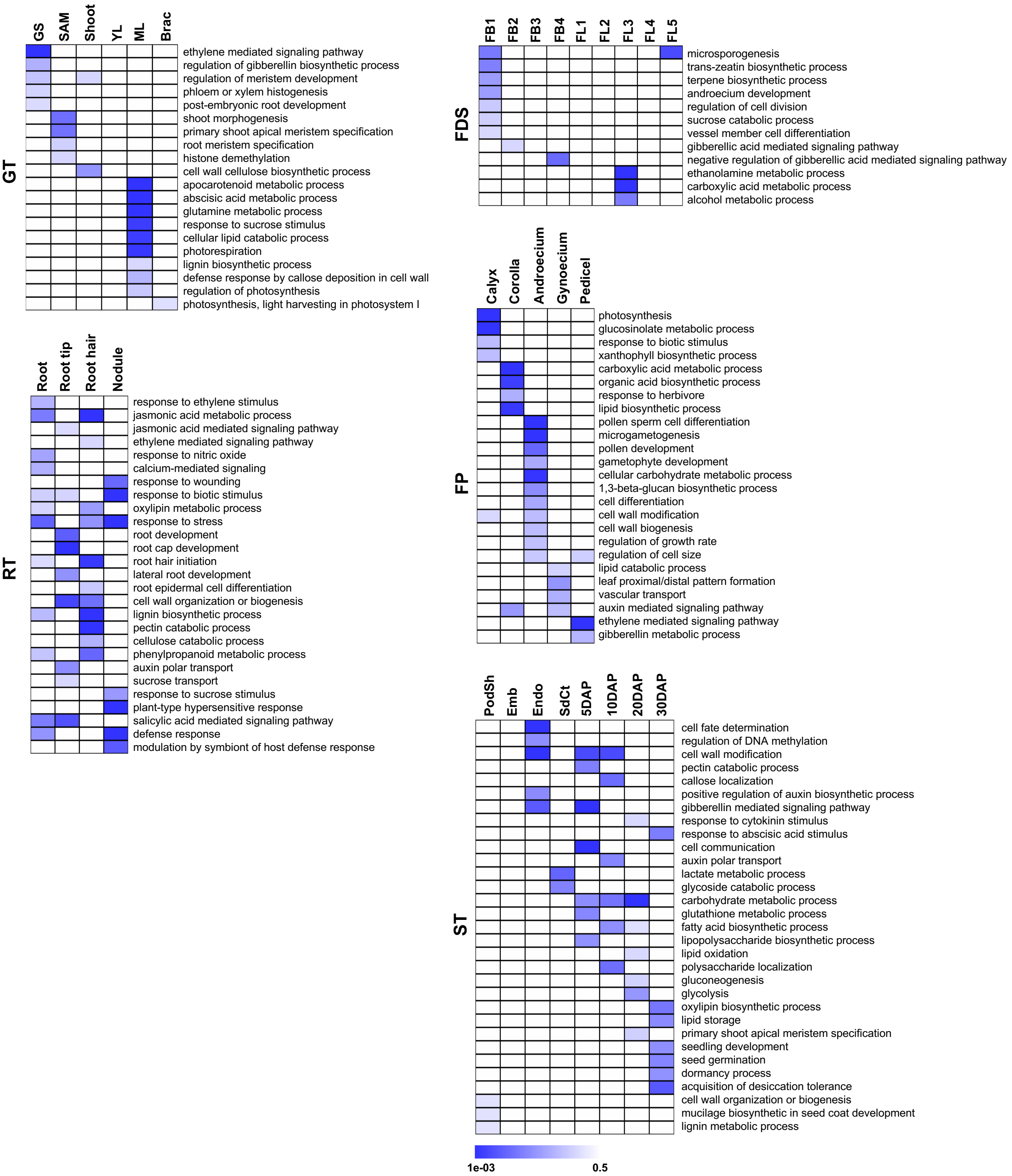

Supplementary Figure 15. GO enrichment analysis of the tissue-specific protein-coding genes identified in the five groups of tissues, including green tissues (GT), root tissues (RT), flower development stages (FDS), flower parts (FP), and seed tissues (ST). Color scale at the bottom represents $\log_{10}$ P-value significance.

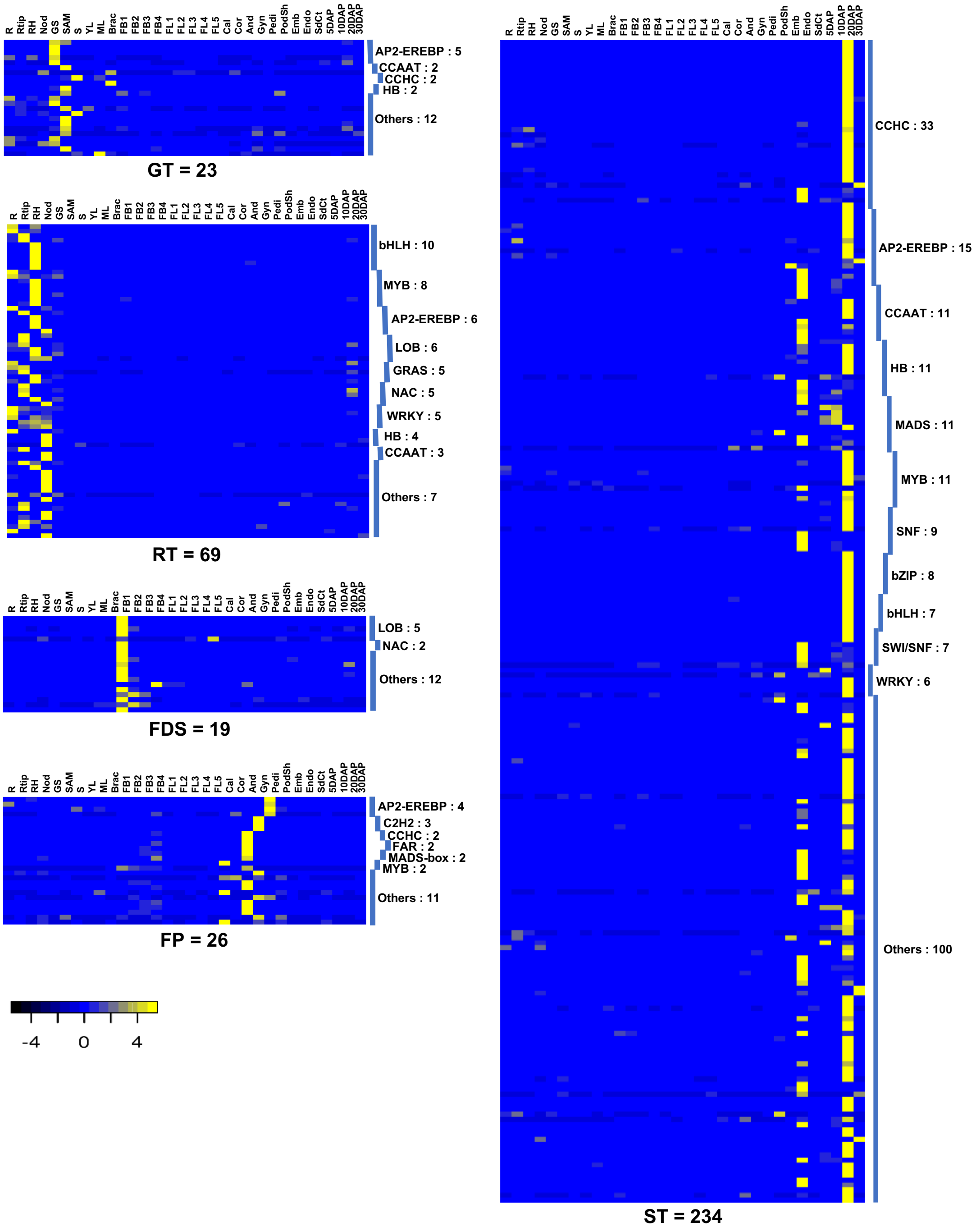

Supplementary Figure 16. Heatmaps showing tissue-specific expression of TF-encoding genes belonging to different families. The number of TFs belonging to different families showing tissue-specific expression in different groups, including green tissues (GT), root tissues (RT), flower development stages (FDS), flower parts (FP), and seed tissues (ST) are given on the right side. Color scale at the bottom represents Z-score.

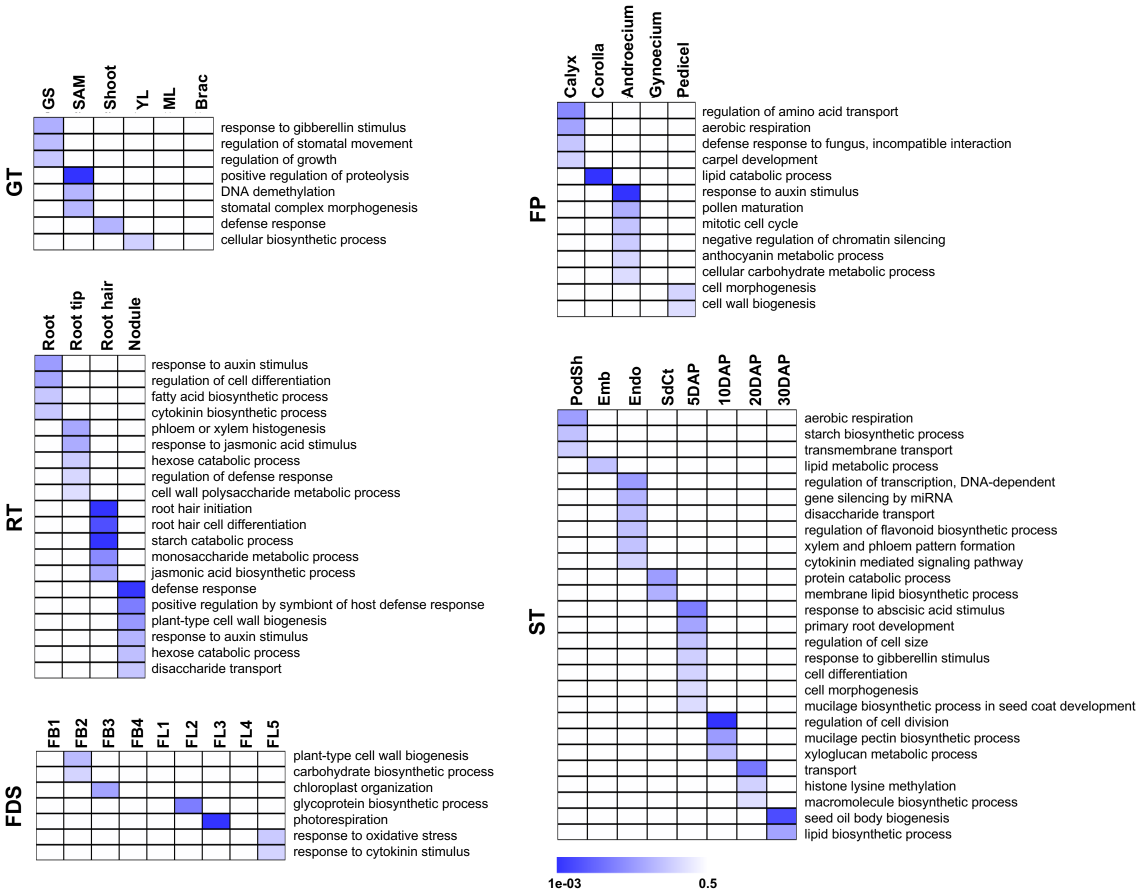

Supplementary Figure 17. GO enrichment analysis of the tissue-specific lncRNAs identified in the five groups of tissues, including green tissues (GT), root tissues (RT), flower development stages (FDS), flower parts (FP), and seed tissues (ST). Color scale at the bottom represents $\log_{10}$ P-value significance.

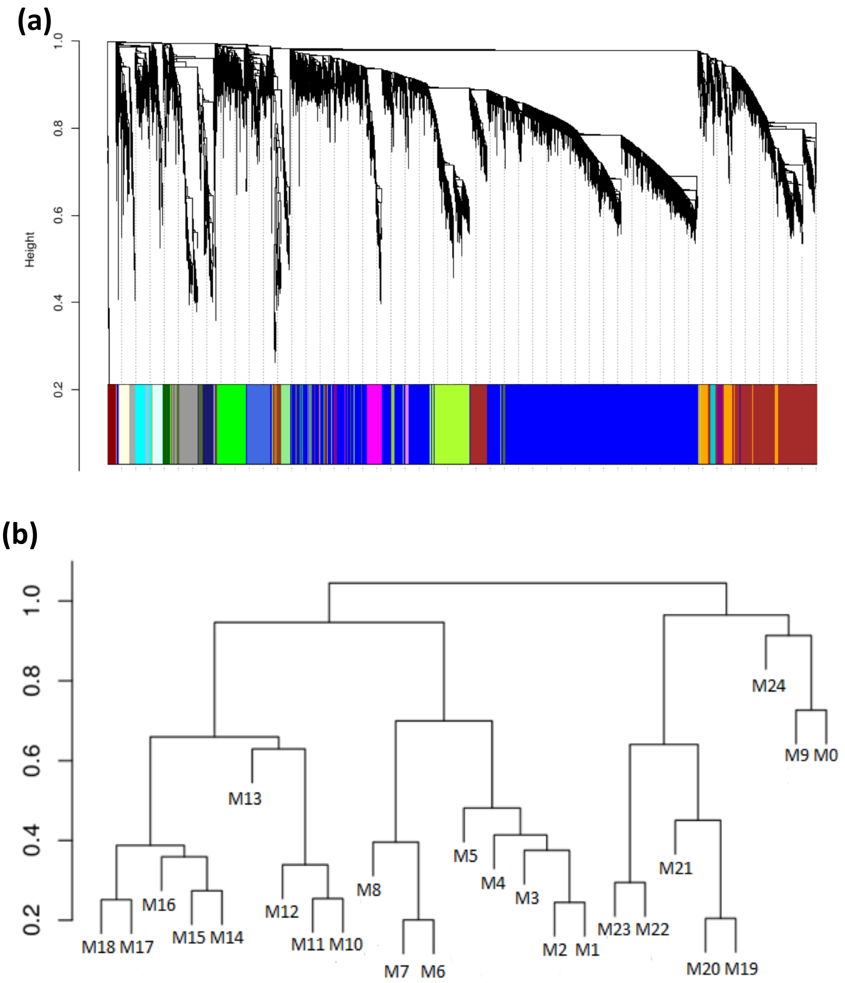

Supplementary Figure 18. Coexpression network analysis in chickpea. (a) Hierarchical clustering tree of transcripts based on coexpression network analysis in chickpea. The transcripts (each short vertical line represents individual transcript) were clustered on the basis of dissimilarity measure. The colored heatmap below the dendrogram indicates the module membership. (b) Dendrogram showing correlation between the identified modules.

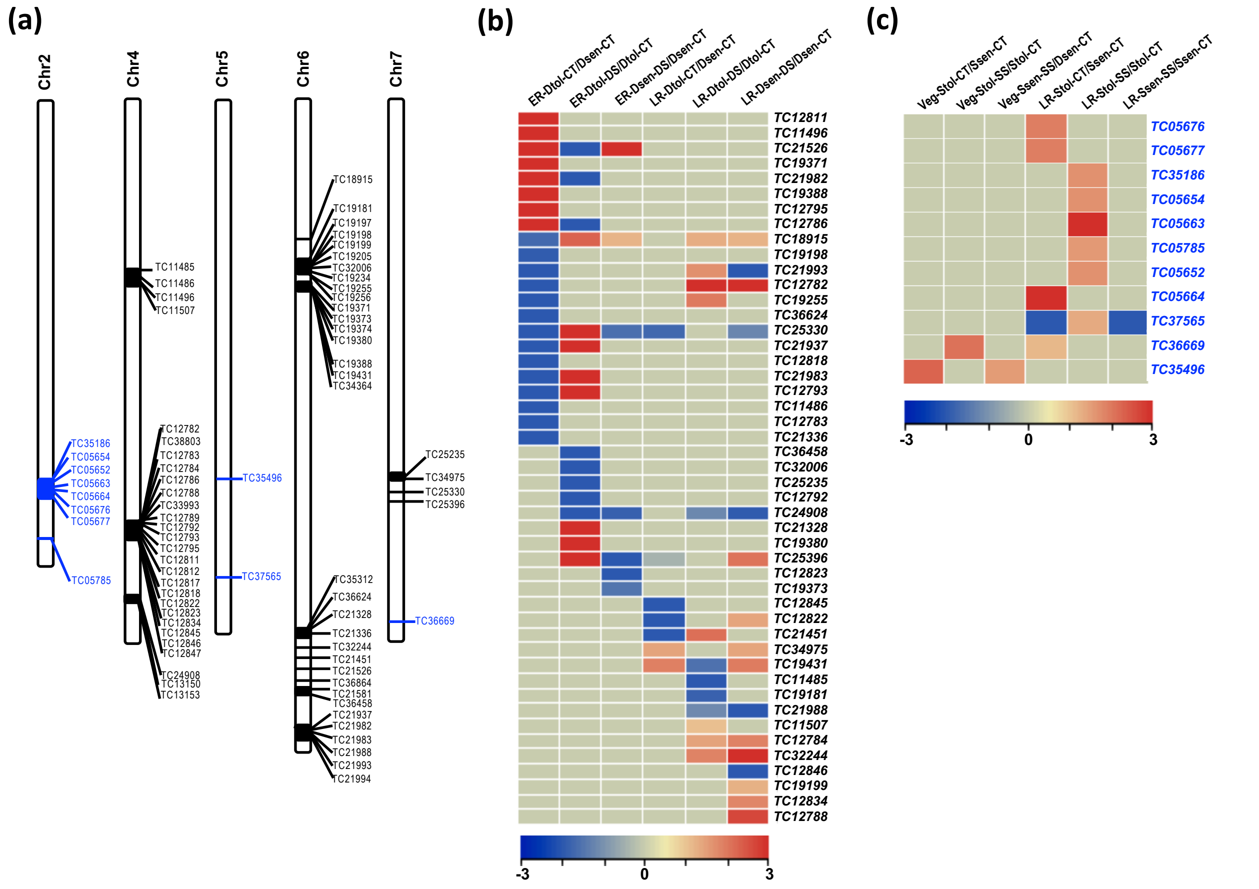

Supplementary Figure 19. Transcripts located in the drought and salinity stress related QTL regions that are differentially expressed under drought and salinity stress conditions. (a) Ideogram depicts the transcripts located in the drought (black) and salinity (blue) related known QTL regions on different chickpea chromosomes harboring DNA polymorphisms within the transcript and or promoter regions and show differential expression under drought and/or salinity stress conditions. (b, c) Heatmaps depict the differential expression of the transcripts harboring DNA polymorphisms within the transcript regions under drought (b) and salinity (c) stress conditions in the chickpea genotypes. Scales at the bottom represent $\log_2$ fold-change differential expression. The differential expression in drought-tolerant (Dtol) and drought-sensitive (Dsen) chickpea genotypes under drought stress (DS) or control (CT) condition at early reproductive (ER) and late reproductive (LR) stages is shown in b. The differential expression in salinity-tolerant (Stol) and salinity-sensitive (Ssen) chickpea genotypes under salinity stress (SS) or control (CT) condition at vegetative (Veg) and late reproductive (LR) stages is shown in c.

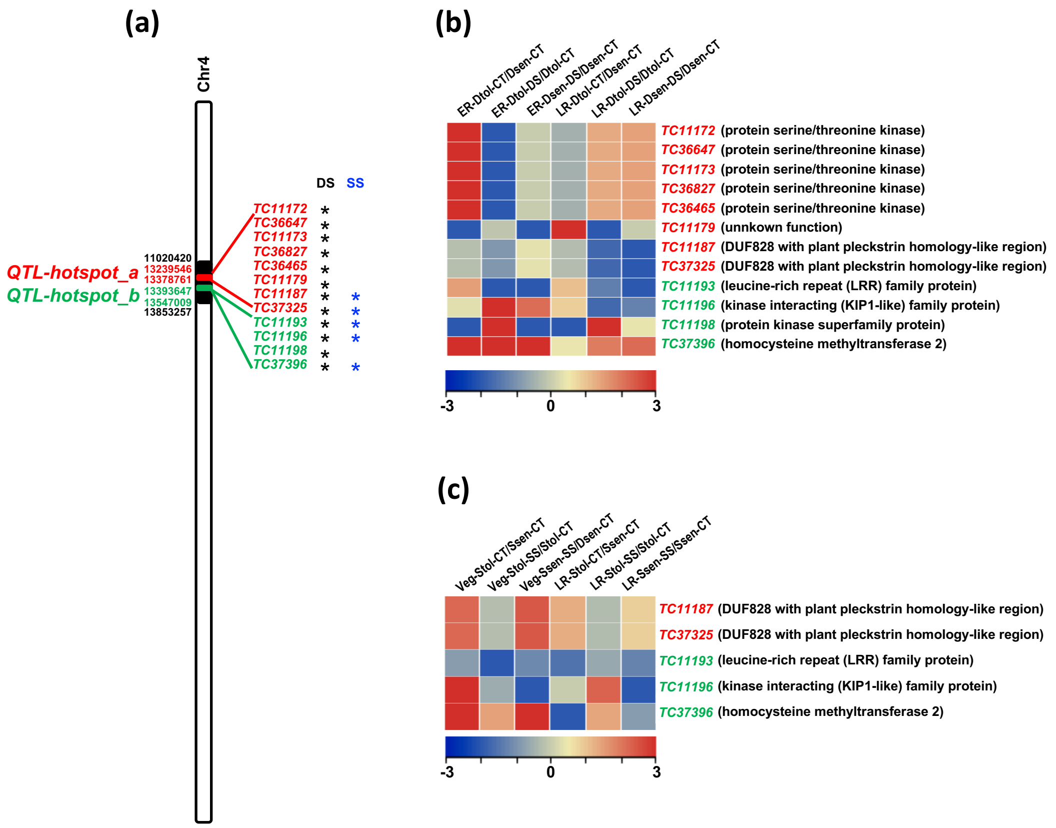

Supplementary Figure 20. Transcripts located in the refined *QTL-hotspot* subregions that are differentially expressed under drought and salinity stress conditions. (a) Ideogram depicts the transcripts located in the refined *QTL-hotspot* subregions (a and b indicated in red and green colors, respectively) on chromosome 4 that are differentially expressed under drought and/or salinity stress conditions. (b, c) Heatmaps depict differential expression of the transcripts given in a under drought (b) and salinity (c) stress conditions. Scales at the bottom represent log_2 fold-change differential expression. The differential expression in drought-tolerant (Dtol) and drought-sensitive (Dsen) chickpea genotypes under drought stress (DS) or control (CT) condition at early reproductive (ER) and late reproductive (LR) stages is shown in b. The differential expression in salinity-tolerant (Stol) and salinity-sensitive (Ssen) chickpea genotypes under salinity stress (SS) or control (CT) condition at vegetative (Veg) and late reproductive (LR) stages is shown in c.
