# Supplementary material for: An integrated transcriptome mapping the regulatory network of coding and long non-coding RNAs provides a genomics resource in chickpea
Source: Commun Biol. 2022 Oct 19;5:1106. doi: 10.1038/s42003-022-04083-4 (PMC9581958; doi:10.1038/s42003-022-04083-4)
Supplement: Supplementary file 3 — Description of Additional Supplementary Data [file 42003_2022_4083_MOESM3_ESM.docx]

**Description of Additional Supplementary Files**

**File name:** Supplementary Data 1

**Description:** Summary of RNA sequencing data generated for different tissue samples using Illumina platform and mapping to the final consensus reference transcriptome assembly.

**File name:** Supplementary Data 2

**Description:** The source data behind the graphs in different figures in the paper.

**File name:** Supplementary Data 3

**Description:** List of transcripts not aligned to the reference chickpea.

**File name:** Supplementary Data 4

**Description:** Functional annotation of protein-coding genes and lncRNAs.

**File name:** Supplementary Data 5

**Description:** FPKM matrix showing expression levels of all the protein-coding genes and lncRNAs in different tissues/organs.

**File name:** Supplementary Data 6

**Description:** Tissue-specificity of protein-coding genes and lncRNAs.

**File name:** Supplementary Data 7

**Description:** Coexpression module membership of protein-coding genes and lncRNAs.

**File name:** Supplementary Data 8

**Description:** List of candidate transcripts showing tissue-specific expression located within QTLs associated with different traits.

**File name:** Supplementary Data 9

**Description:** Transcripts harboring DNA polymorphisms (within transcript and/or promoter regions).

**File name:** Supplementary Data 10

**Description:** List of candidate transcripts showing differential expression under the drought stress harboring DNA polymorphisms within the transcript and/or their promoter regions.

**File name:** Supplementary Data 11

**Description:** Differential expression of transcripts harboring DNA polymorphisms.
