# Supplementary material for: An integrated transcriptome mapping the regulatory network of coding and long non-coding RNAs provides a genomics resource in chickpea
Source: Commun Biol. 2022 Oct 19;5:1106. doi: 10.1038/s42003-022-04083-4 (PMC9581958; doi:10.1038/s42003-022-04083-4)
Supplement: Supplementary file 15 — Reporting Summary [file 42003_2022_4083_MOESM15_ESM.pdf]

nature research

Corresponding author(s): M. Jain

Last updated by author(s): Jul 8, 2021

Reporting Summary

Nature Research wishes to improve the reproducibility of the work that we publish. This form provides structure for consistency and transparency in reporting. For further information on Nature Research policies, see Authors & Referees and the Editorial Policy Checklist.

Statistics

For all statistical analyses, confirm that the following items are present in the figure legend, table legend, main text, or Methods section.

n/a	Confirmed	
☐	☒	The exact sample size (n) for each experimental group/condition, given as a discrete number and unit of measurement
☐	☒	A statement on whether measurements were taken from distinct samples or whether the same sample was measured repeatedly
☐	☒	The statistical test(s) used AND whether they are one- or two-sided<br>*Only common tests should be described solely by name; describe more complex techniques in the Methods section.*
☒	☐	A description of all covariates tested
☐	☒	A description of any assumptions or corrections, such as tests of normality and adjustment for multiple comparisons
☐	☒	A full description of the statistical parameters including central tendency (e.g. means) or other basic estimates (e.g. regression coefficient) AND variation (e.g. standard deviation) or associated estimates of uncertainty (e.g. confidence intervals)
☐	☒	For null hypothesis testing, the test statistic (e.g. F, t, r) with confidence intervals, effect sizes, degrees of freedom and P value noted<br>*Give P values as exact values whenever suitable.*
☒	☐	For Bayesian analysis, information on the choice of priors and Markov chain Monte Carlo settings
☒	☐	For hierarchical and complex designs, identification of the appropriate level for tests and full reporting of outcomes
☒	☐	Estimates of effect sizes (e.g. Cohen's d, Pearson's r), indicating how they were calculated

Our web collection on statistics for biologists contains articles on many of the points above.

Software and code

Policy information about availability of computer code

Data collection	Data was obtained after sequencing of the total RNA samples.
Data analysis	Only open source software were used. All softwares used are listed along with their versions in the Methods section. For example, The raw reads were processed to remove reads with adaptor sequences and low-quality reads using NGSQC Toolkit (v2.3). The high-quality filtered reads were mapped to chickpea transcriptome assembly using Bowtie (v2.3.4.1) and gene expression was quantified using RSEM (v1.2.28). Enrichment of GO terms was carried out using BiNGO tool at Cytoscape (v3.7). Gene coexpression network analysis was performed using weighted gene co-expression network analysis (WGCNA v1.61) package. Enrichment of sequence motifs was performed using HOMER (v4.9.1).

For manuscripts utilizing custom algorithms or software that are central to the research but not yet described in published literature, software must be made available to editors/reviewers. We strongly encourage code deposition in a community repository (e.g. GitHub). See the Nature Research guidelines for submitting code & software for further information.

Data

Policy information about availability of data

All manuscripts must include a data availability statement. This statement should provide the following information, where applicable:

- Accession codes, unique identifiers, or web links for publicly available datasets
- A list of figures that have associated raw data
- A description of any restrictions on data availability

The PacBio sequencing data generated in this study are available in the SRA public repository under the bioproject accession number PRJNA613159. Illumina RNA sequencing data and the reference transcriptome assembly generated in this study have been deposited in the Gene Expression Omnibus (GEO) database under the series accession number GSE147831.Different datasets generated in this study, including reference transcriptome assembly, annotation and expression data are available for bulk download via the webpage http://ccbb.jnu.ac.in/CaGEA. The different annotation and expression data are provided in the Supplemental Data files too.

Field-specific reporting

Please select the one below that is the best fit for your research. If you are not sure, read the appropriate sections before making your selection.

☒ Life sciences ☐ Behavioural & social sciences ☐ Ecological, evolutionary & environmental sciences

For a reference copy of the document with all sections, see nature.com/documents/nr-reporting-summary-flat.pdf

Life sciences study design

All studies must disclose on these points even when the disclosure is negative.

Sample size	No sample-size calculation was performed. For each tissue/developmental stage, samples from different plants were pooled to make each biological replicate.
Data exclusions	No data exclusions were done.
Replication	Three biological replicates of most of the samples were used for sequencing (RNA sequencing) for each tissue/stage of development analyzed.
Randomization	Different samples were grouped according to the pre-defined tissue/development stage.
Blinding	Blinding was not relevant with the data present in this study. Sequencing data for each pre-defined tissue/developmental stage sample were collected and analyzed separately.

Reporting for specific materials, systems and methods

We require information from authors about some types of materials, experimental systems and methods used in many studies. Here, indicate whether each material, system or method listed is relevant to your study. If you are not sure if a list item applies to your research, read the appropriate section before selecting a response.

Materials & experimental systems

n/a	Involved in the study	
☒	☐	Antibodies
☒	☐	Eukaryotic cell lines
☒	☐	Palaeontology
☒	☐	Animals and other organisms
☒	☐	Human research participants
☒	☐	Clinical data

Methods

n/a	Involved in the study	
☒	☐	ChIP-seq
☒	☐	Flow cytometry
☒	☐	MRI-based neuroimaging
